# Resident vs nonresident multipotent mesenchymal stromal cell interactions with B lymphocytes result in disparate outcomes

**DOI:** 10.1002/sctm.20-0289

**Published:** 2021-01-28

**Authors:** Wei Lee, Li‐Tzu Wang, Men‐Luh Yen, Pei‐Ju Hsu, Yu‐Wei Lee, Ko‐Jiunn Liu, Kuo‐I Lin, Yu‐Wen Su, Huey‐Kang Sytwu, B. Linju Yen

**Affiliations:** ^1^ Graduate Institute of Life Sciences, National Defense Medical Center (NDMC) Taipei Taiwan; ^2^ Regenerative Medicine Research Group Institute of Cellular and System Medicine, National Health Research Institutes (NHRI) Zhunan Taiwan; ^3^ Department of Obstetrics/Gynecology National Taiwan University (NTU) Hospital and College of Medicine, NTU Taipei Taiwan; ^4^ National Institute of Cancer Research, NHRI Tainan Taiwan; ^5^ Genomics Research Center Academia Sinica Taipei Taiwan; ^6^ Immunology Research Center NHRI Zhunan Taiwan; ^7^ National Institute of Infectious Diseases & Vaccinology, NHRI Zhunan Taiwan; ^8^ Graduate Institute of Microbiology & Immunology NDMC Taipei Taiwan

**Keywords:** bone marrow (BM), human mesenchymal stromal cells (MSCs), interleukin‐10 (IL‐10), peripheral B lymphocytes, placenta, regulatory B cells (Bregs), tissue specificity

## Abstract

Multipotent human mesenchymal stromal cells (MSCs) from multiple organs including the bone marrow (BM) and placenta harbor clinically relevant immunomodulation best demonstrated toward T lymphocytes. Surprisingly, there is limited knowledge on interactions with B lymphocytes, which originate from the BM where there is a resident MSC. With increasing data demonstrating MSC tissue‐specific propensities impacting therapeutic outcome, we therefore investigated the interactions of BM‐MSCs—its resident and “niche” MSC—and placental MSCs (P‐MSCs), another source of MSCs with well‐characterized immunomodulatory properties, on the global functional outcomes of pan‐peripheral B cell populations. We found that P‐MSCs but not BM‐MSCs significantly inhibit proliferation and further differentiation of stimulated human peripheral B populations in vitro. Moreover, although BM‐MSCs preserve multiple IL‐10‐producing regulatory B cell (Breg) subsets, P‐MSCs significantly increase all subsets. To corroborate these in vitro findings in vivo, we used a mouse model of B‐cell activation and found that adoptive transfer of P‐MSCs but not BM‐MSCs significantly decreased activated B220^+^ B cells. Moreover, adoptive transfer of P‐MSCs but not BM‐MSCs significantly decreased the overall B220^+^ B‐cell proliferation and further differentiation, similar to the in vitro findings. P‐MSCs also increased two populations of IL‐10‐producing murine Bregs more strongly than BM‐MSCs. Transcriptome analyses demonstrated multifactorial differences between BM‐ and P‐MSCs in the profile of relevant factors involved in B lymphocyte proliferation and differentiation. Our results highlight the divergent outcomes of tissue‐specific MSCs interactions with peripheral B cells, and demonstrate the importance of understanding tissue‐specific differences to achieve more efficacious outcome with MSC therapy.

## Graphical abstract

Multipotent bone marrow mesenchymal stromal cells (BM‐MSCs)—the resident MSC for B lymphocyte development—support stimulated peripheral B‐cell proliferation/differentiation, whereas placental MSCs (P‐MSCs) suppress proliferation/differentiation and more significantly increased multiple populations of IL‐10‐expressing regulatory B cells (Bregs). These divergent outcomes are likely due to multifactorial differences in the expression of relevant factors by the two MSC sources.

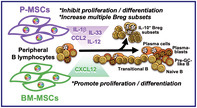

**Significance statement**

Human multilineage mesenchymal stem cells (MSCs) harbor clinically relevant immunomodulation, but studies on B cells are surprisingly rare. This study therefore investigated the in vitro and in vivo interactions of pan‐peripheral B lymphocyte populations with bone marrow (BM) MSCs—its resident and “niche” MSC—and placental MSCs (P‐MSCs), another source of MSCs with well‐characterized immunomodulatory properties. This study found that BM‐ and P‐MSCs differentially modulate B‐cell proliferation and differentiation, including multiple IL‐10‐producing regulatory B populations, which are likely due to multifactorial differences in the profile of relevant factors expressed by the two types of MSCs. The results highlight the importance of understanding tissue‐specific differences to achieve more efficacious outcome with MSC therapy.

## 1 INTRODUCTION

Mesenchymal stromal cells (MSCs) are multipotent somatic progenitor cell with strong immunomodulatory properties which have been best reported toward T lymphocytes.^1^ First isolated from the bone marrow (BM), these somatic progenitor cells have subsequently been found to exist in numerous other adult as well as fetal‐derived organs/tissues, with most of these sources amenable to clinical use.^2^, ^3^ However, definitive clinical success has proven difficult to achieve, and it is now believed that important functional differences exist between MSCs isolated from different sources/tissue which have therapeutic ramifications.^4^, ^5^, ^6^ Thus, more research is urgently warranted in this area.

Responsible for the humoral immunity in adaptive immunity, B lymphocytes are a critical component of the immune system and yet there is surprising little known about their interactions with MSCs. Moreover, although both B and T lymphocytes originate from the hematopoietic stem cells residing in the BM, T cells migrate to the thymus for further development whereas B cells remain in the BM where there is a resident MSCs: the BM‐MSCs. As immature B cells, these lymphocytes then leave the BM and migrate into secondary lymphocyte tissues of the spleen and lymph nodes, where the majority undergo the conventional maturation and differentiation pathway into memory B cells and antibody‐secreting cells of plasmablasts and plasma cells.^7^, ^8^ This well‐known paradigm of B‐cell development has increasingly been expanded in recent years, with discoveries of alternative differentiation pathways into immunomodulatory B cells—IL‐10‐secreting B regulatory cells (Bregs)^9^—as well as identification of crucial B‐cell roles in diseases traditionally viewed not to involve these cells, including numerous autoimmune diseases and even cancer.^10^, ^11^ Such studies implicate the important influence that microenvironments have on peripheral B‐cell differentiation and maturation trajectories. Hence, there has been a resurgence of interest to better understand the complex landscape of peripheral B‐cell subsets and functions.

In contrast to MSC T‐cell interactions, studies on MSC‐B cell interactions are scarce with less than 40 publications and show discrepant results.^12^ The majority of these studies use BM‐MSCs, with both promotion^13^, ^14^, ^15^ and inhibition^16^, ^17^, ^18^, ^19^ of B‐cell growth reported. The most consistent factor reported in these studies is B‐cell activating factor, but there is still disagreement on whether BM‐MSCs upregulate^13^, ^15^, ^20^ or downregulate^18^, ^21^ this protein. Disparate results are also found regarding B‐cell antibody secretion, with both increases^13^, ^14^ and decreases^17^, ^18^, ^22^ reported. Clearly, the increasing relevance of MSC therapy for immune‐related diseases and the involvement of B cells in these diseases argues for more in‐depth investigation. We therefore assessed the interactions of peripheral B lymphocytes to two well‐studied sources of human MSCs with strong immunomodulatory properties reported toward T cells and myeloid cells^1^, ^23^, ^24^: adult BM‐MSCs, the resident native‐niche MSCs for B cells, and placenta‐derived MSCs (P‐MSCs), which are isolated from an ethically compliant and immunomodulatory organ without requiring invasive procedures.^25^ P‐MSCs have been demonstrated to be fetal in origin, and thus have additional advantages of high cell yield and some intrinsic immunomodulatory capacity which may not require inflammatory priming.^26^, ^27^, ^28^, ^29^ We therefore evaluated the interactions of both types of MSCs against human and mouse B lymphocytes in in vitro and in vivo studies, respectively, and performed transcriptome profiling to delineate factor(s) involved in these interactions.

## 2 MATERIALS AND METHODS

### 2.1 Cell culture

Human MSCs from BM and placenta were cultured and expanded as we previously described.^25^, ^26^ P‐MSCs were isolated as previously reported from the term placentae (38‐40 weeks of gestation) of healthy donor mothers obtained with informed consent according to the procedures of the institutional review board.^25^ Characterized human BM‐MSCs were purchased and cultured according to manufacturer's instructions (PromoCell, Heidelberg, Germany). Both types of MSCs conform to the minimal criteria for multipotent MSCs including surface marker expression profile and differentiation into osteoblastic, adipocytic, and chondrocytic lineages.^24^, ^25^, ^26^, ^27^, ^29^, ^30^ P‐MSCs between the passages of 15 and 20 and BM‐MSCs between the passages of 8 and 10 were used.^29^

For B lymphocyte isolation and activation, peripheral blood mononuclear cells (PBMCs) were isolated from human buffy coats of healthy donor blood samples (Taiwan Blood Services Foundation, Taipei Blood Center, Taipei, Taiwan) obtained with informed consent according to the procedures of the institutional review board. After PBMC isolation by Ficoll‐Paque (1.077 g/mL; Thermo Fisher Scientific, Waltham, Massachusetts) density gradient centrifugation, B cells were enriched by negative selection using EasySep Human Pan‐B Cell Enrichment kit (STEMCELL Technologies, Vancouver, British Columbia, Canada)^31^ according to the manufacturer's protocol. The resulting cell fraction contained >90% CD19^+^ B cells. The contaminant cells were mainly T cells (1.9% ± 0.7%) with <0.5% for other cell types. After the enrichment, B cells were cultured in Iscove's Modified Dulbecco's Medium containing 10% FCS, 1 mM sodium pyruvate, 100 U/mL penicillin, 100 g/mL streptomycin, and 2 mM L‐glutamine (B cell medium). An activation cocktail to mimic antigen‐ and T cell‐mediated stimulation was used as previously described with minor modifications^32^: F(ab)2 anti‐IgM (5 μg/mL; Jackson ImmunoResearch, West Grove, Pennsylvania), anti‐CD40 agonistic monoclonal antibody (0.5 μg/mL; BioLegend, San Diego, California), toll‐like receptor (TLR) activation CpG (2.5 μg/mL; Thermo Fisher Scientific), and IL‐2 (100 IU; Thermo Fisher Scientific). For coculture experiments, B cells were first labeled with carboxyfluorescein succinimidyl ester (CFSE, Invitrogen, Carlsbad, California) and cocultured with MSCs at a ratio of 10:1 (2 × 10^5^ B cells with 2 × 10^4^ MSCs) in 96‐well plates and B cell medium. Cell counting was performed using Bürker chamber after staining with trypan blue. Cells were then collected for flow cytometric analysis (FACSVerse, FACSuite Software; BD Biosciences, San Jose, California) and analyzed by FlowJo (BD Biosciences).

### 2.2 Flow cytometry analysis

Single‐cell suspensions were stained with a mixture of 5 to 6 fluorophores including and 7‐aminoactinomycin D (7‐AAD) for viability and acquired on the BD Verse flow cytometer with data analyzed using FlowJo software (BD Biosciences). Human B‐cell subsets were identified using the following antibodies: CD19‐APC, CD27‐BV510, CD38‐APC‐Cy7, CD24‐BV421, and IL‐10‐PE‐Cy7 (all from BioLegend). Mouse B‐cell subsets were identified using the following antibodies: B220‐PE, CD23‐APC, CD21‐PE‐Cy7, IgM‐BV421, CD138‐APC, CD86‐PE‐Cy7, CD69‐APC‐Cy7, and IL‐10‐APC‐Cy7 (all from BioLegend). Intracellular IL‐10 expression was detected as previously reported.^33^ Immunophenotype of human and murine B‐cell subsets analyzed are listed in Supplementary Table 1.

### 2.3 T‐distributed stochastic neighbor embedding (tSNE)‐based analysis

tSNE was analyzed by FlowJo and performed using default parameters (iterations = 500, perplexity = 20, *θ* = 0.5). Samples were randomly downsampled to 2000 to 6000 events per sample. The range in events was determined by the sample with the fewest events acquired and analysis was run on equal numbers of events per sample. Individual flow cytometry sample files were combined into a single flow cytometry standard file to assist in defining spatially distinct populations using the concatenation tool.

### 2.4 In vivo B‐cell stimulation and adoptive transfer of human MSCs

All animal work was performed according to protocols approved by the Institutional Animal Care and Use Committee. Wild type 6 to 8 week‐old C57BL/6J mice were purchased from the National Laboratory Animal Center (Taipei, Taiwan) and induction of in vivo stimulation was performed as previously reported.^34^, ^35^, ^36^ Briefly, lipopolysaccharide (LPS; 100 μg; *Escherichia coli* 00041:B4; Sigma‐Aldrich, Saint Louis, Missouri) was injected intraperitoneally into mice followed 2 hours later by transfer of human MSCs (1 × 10^5^ cells).^35^ MSCs were resuspended in PBS, and the PBS was used as the control. Mice were sacrificed on day 3 with excision of spleen and harvesting of splenocytes for flow cytometric analysis of B‐cell subsets.

### 2.5 Analyses of cDNA microarray

Total RNA was isolated from cells using RNeasy Mini Kits (Qiagen, Hilden, Germany). The RNA samples were used for cDNA synthesis when the A260/280 ratio was assessed to be greater than 1.9. The Affymetrix cDNA microarray hybridization and analysis were performed on GeneChip Human Transcriptome Array 2.0 by the NHRI Microarray Core (Zhunan, Taiwan).^36^ All raw data can be accessed at the National Center for Biotechnology Information‐Gene Expression Omnibus (NCBI‐GEO): GSE101473.

### 2.6 Statistical analyses

Analyses were performed using GraphPad Prism software (La Jolla, California), and data are represented as mean ± SD. Differences were determined using ANOVA with Bonferroni's correction for multiple groups. A value of *P* < .05 defined as statistically significant.

## 3 RESULTS

### 3.1 P‐MSCs but not BM‐MSCs significantly inhibit proliferation and further differentiation of multiple human peripheral B‐cell subsets in vitro

**FIGURE 1:**
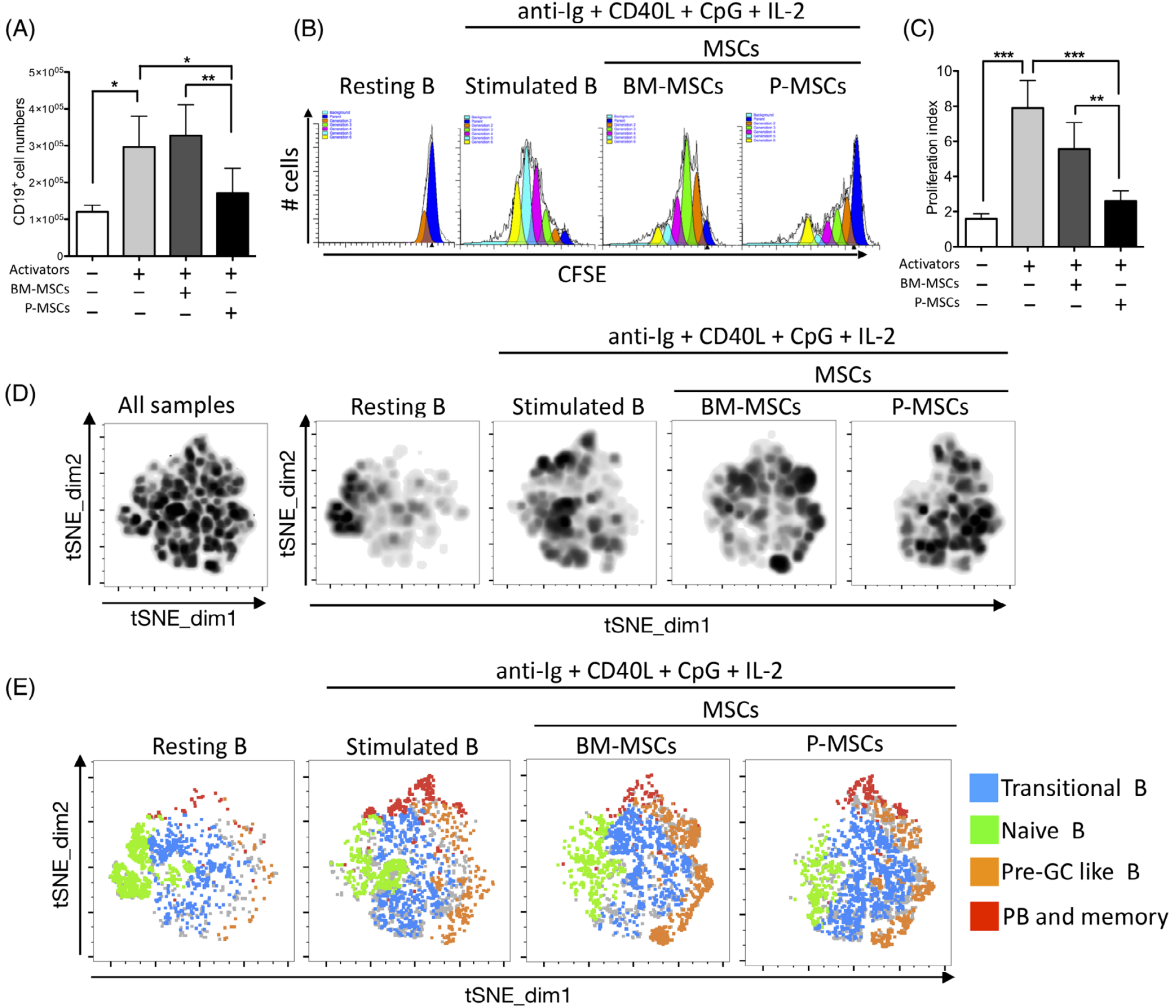
Human placenta mesenchymal stromal cells (P‐MSCs) but not bone marrow (BM‐) MSCs inhibit stimulated B‐cell proliferation significantly and maintained immature transitional B‐cell subset in vitro. A, Pooled data of cell numbers of human CD19^+^ peripheral pan‐B cell (8 healthy donors) stimulated with a cocktail of activators consisting of F(ab)_2_ anti‐IgM, anti‐CD40 agonistic monoclonal antibody, toll‐like receptor (TLR) activation CpG‐ODNs, and IL‐2 with or without coculture of human BM‐MSCs (three donors) or P‐MSCs (three donors) for 6 days with subsequent analysis by flow cytometry. B, Representative data and, C, pooled data of B‐cell proliferation as assessed by staining with CFSE after coculture with either BM‐ or P‐MSCs to monitor distinct generations of proliferating B cells by flow cytometric analyses using ModFit LT software to generate proliferation index data. D, T‐distributed stochastic neighbor embedding (tSNE) plots for analysis of changes in human peripheral B‐cell subsets (five healthy donors) after coculture with either BM‐ or P‐MSCs, as visualized by density plot of concatenated flow cytometry standard files for all samples, and individual sample file for resting B, stimulated B, coculture with BM‐MSCs (three donors), or P‐MSCs (three donors). tSNE analysis was run on 2000 live CD19+ single cells per sample. E, Overlay of manually gated cell populations on to tSNE plots. B‐cell subsets are identified using flow cytometric biaxial gating strategies based on four surface markers into transitional B (CD19^+^ CD27^−^ CD38^hi^ CD24^hi^), naïve B (CD19^+^ CD27^−^ CD38^−/int^ CD24^−/int^), pre‐GC‐like B (CD19^+^ CD27^−^ CD38^hi^ CD24^−/int^), and plasmablast/memory B (CD19^+^ CD27^+^). All samples = 80 000 events; individual resting B and stimulated B samples = 10 000 events (n = 5); individual MSCs cocultured samples = 30 000 events (n = 15). Data are shown as mean ± SD. *P < .05; **P < .01; ***P < .001

To investigate interactions of peripheral B cells globally with resident vs nonresident MSCs, we isolated pure pan‐B population from human PBMCs by negative‐selection magnetic beads to capture all B‐cell subsets, including naïve B, memory B, and plasma cells.^31^ Using a cocktail of anti‐Ig, CD40L, CpG‐ODNs, and IL‐2 to stimulate resting B cells,^12^ we found that B‐cell differentiation was most robust on day 6 as evidenced by abundant IgG^+^ plasmablast percentages (Figure S1); based on this data, all in vitro experiments were carried out for 6 days. We then performed coculture of cocktail‐stimulated B cells with either BM‐ or P‐MSCs, and found B‐cell proliferation to be significantly inhibited down to nearly baseline/unstimulated levels with P‐MSC but not BM‐MSC coculture (Figure 1A). Using CFSE to monitor distinct generations of proliferating cells (Figure 1B, representative data; Figure 1C, pooled data), we found that proliferation of stimulated B cells was significantly inhibited after coculturing with P‐MCSs (proliferation index 2.63 ± 0.4) but not BM‐MSCs (proliferation index 5.09 ± 0.9). Both BM‐ and P‐MSCs globally modulate stimulated peripheral B‐cell subsets but in different trajectories, as evidenced by tSNE analysis using traditional biaxial gating strategies based on four surface markers to identify B‐cell subsets—CD19^+^ CD27^−^ CD38^hi^ CD24^hi^ for transitional B, CD19^+^ CD27^−^ CD38^−/int^ CD24^−/int^ for naïve B, CD19^+^ CD27^−^ CD38^hi^ CD24^−/int^ for pre‐germinal center (GC)‐like B, and CD19^+^ CD27^+^ for plasmablast/memory B—then concatenated. We found that compared with stimulated B cells alone, after coculture with P‐MSCs the transitional B subset scan be more prominently visualized, whereas after coculture with BM‐MSCs the pre‐GC‐like B subset was more prominently visualized (Figure 1D, intensity plot; and Figure 1E, B‐cell subset plot). These findings demonstrate that P‐MSCs inhibit stimulated peripheral B‐cell proliferation and may maintain these lymphocytes in a less differentiated state, whereas in contrast, BMMSCs promote proliferation and may support further differentiation of stimulated peripheral B cells.

**FIGURE 2:**
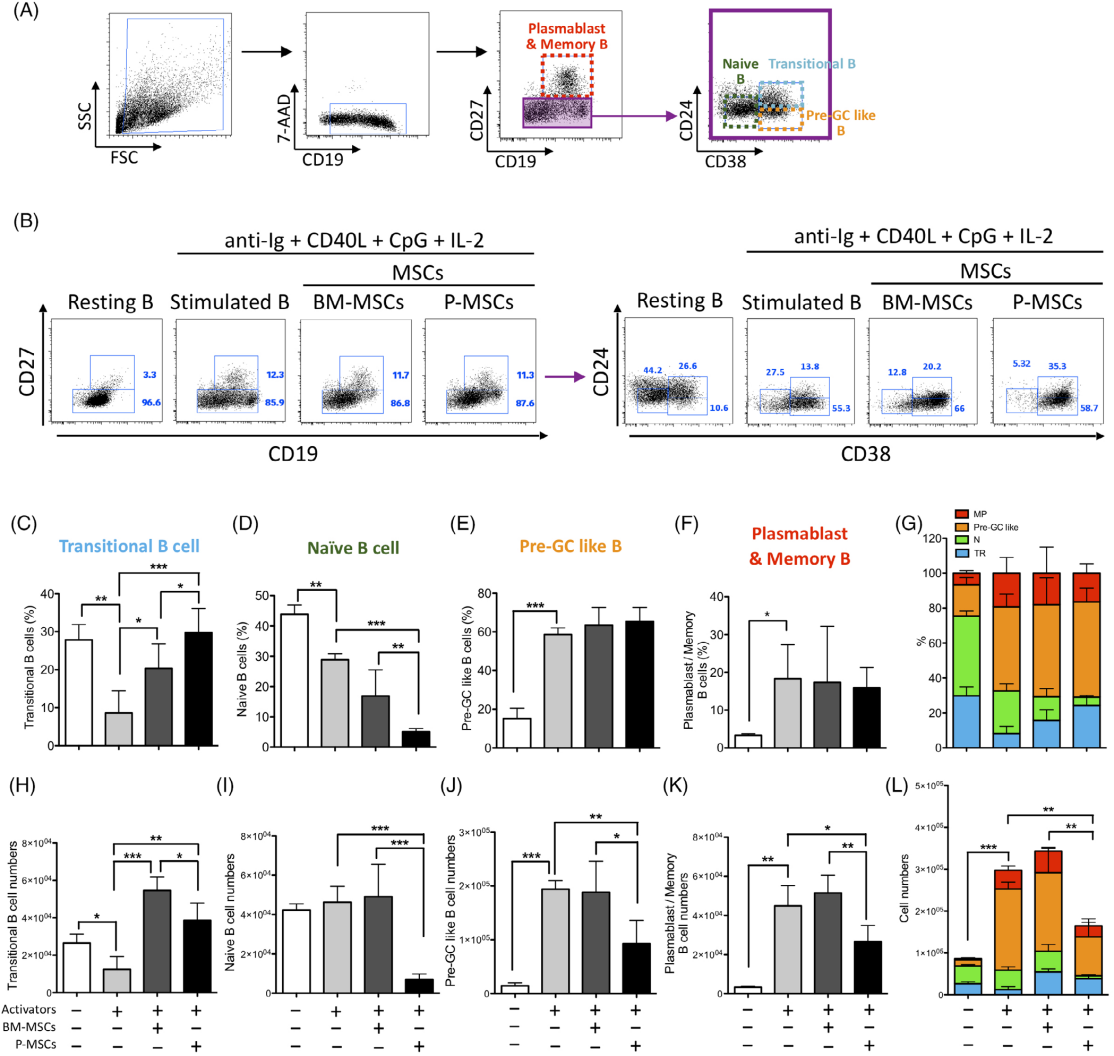
Placenta mesenchymal stromal cells (P‐MSCs) but not bone marrow (BM)‐MSCs significantly inhibited proliferation of multiple human peripheral B‐cell subsets in vitro. A, Flow cytometric gating strategy for identifying B‐cell subsets including transitional B (CD19^+^ CD27^−^ CD38^hi^ CD24^hi^), naive B (CD19^+^ CD27^−^ CD38^−/int^ CD24^−/int^), pre‐GC‐like B (CD19^+^ CD27^−^ CD38^hi^ CD24^−/int^), and plasmablast/memory B (CD19^+^ CD27^+^). B, Representative data for B‐cell subset analyses in resting B cells, and cocktail‐stimulated B cells cocultured without or with either BM‐MSCs (three donors) or P‐MSCs (three donors). B cells were obtained from five healthy donors. Pooled data on subset percentages and absolute cell number are presented, respectively, as follows: (C,H) transitional B, (D,I) naive B, (E,J) pre‐GC‐like B, and (F,K) plasmablasts/memory B; and integrated data of (G) B‐cell subset percentages and (L) absolute cell numbers. MP, plasmablasts and memory B; N, naive B; TR, transitional B. All percentage figures are gated on CD19^+^ cells. Data are shown as mean ± SD. *P < .05; **P < .01; ***P < .001

To further analyze these global changes to major peripheral B‐cell subsets after coculture with BM‐ and P‐MSCs, we quantified for specific changes to subset percentages and absolute cell counts (Figure 2A,B, gating strategy and representative flow cytometric dot plots, respectively). In comparison to coculture with BM‐MSCs, stimulated B cells cocultured with P‐MSCs resulted in further significant expansion of the transitional B subset but contraction of the naive B subset in terms of cell percentages (Figure 2C,D, percentages of transitional B and naive B, respectively); but for pre‐GC like B and plasmablast/memory B subsets, both MSCs exerted similar effects of maintaining these two subset percentages (Figure 2E,F, percentages of pre‐GC like B and plasmablast/memory B, respectively; Figure 2G, integrated subset percentages). However, when absolute cell numbers are examined, the strong suppressive effects of P‐MSCs on stimulated B cells in general are readily apparent: compared to coculture with BM‐MSCs, stimulated B cells cocultured with P‐MSCs resulted in significant suppression of proliferation and cell numbers in all B‐cell subsets (Figure 2H‐K, absolute cell numbers in specific B subsets as denoted; Figure 2L, integrated subset cell numbers; Figure S2, proliferation data for each subset). Closer examination show that the suppressive effects of P‐MSCs was especially strong on the naïve B subset (Figure 2D,I), but weakest on the transitional B subset (Figure 2C,H). Conversely, coculture with BM‐MSCs significantly increased transitional B subset cell counts (Figure 2H) and maintained the cell counts for all other subsets (Figure 2I‐K). Taken together, these findings show that BM‐MSCs support differentiation of stimulated peripheral B‐cell subsets, whereas P‐MSCs significantly suppressed overall stimulated peripheral B‐cell proliferation as well as further differentiation.

### 3.2 P‐MSCs significantly increase multiple populations of IL‐10‐producing regulatory B than BM‐MSCs in vitro

**FIGURE 3:**
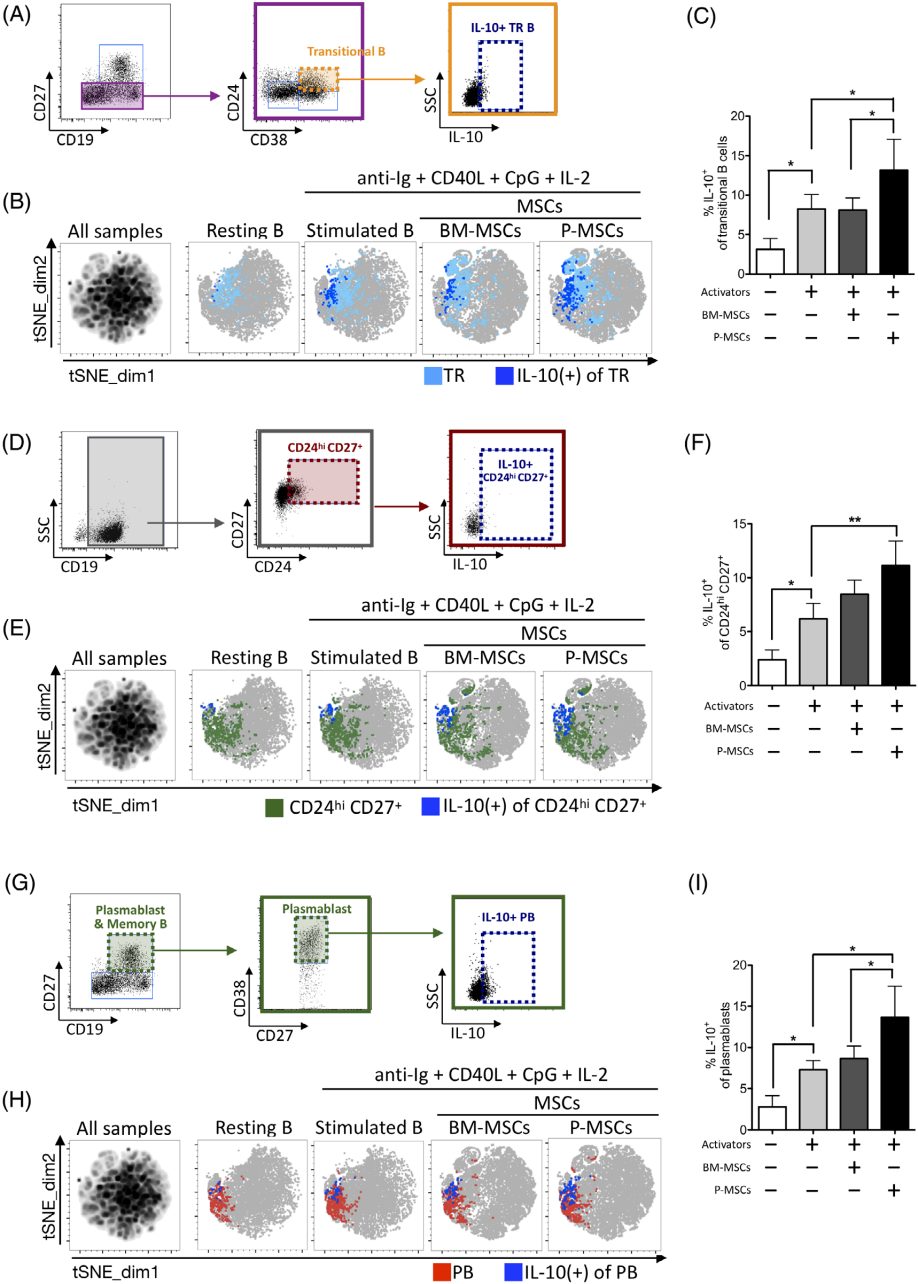
Placenta mesenchymal stromal cells (P‐MSCs) significantly increase multiple populations of IL‐10‐producing regulatory B cells (Bregs) than bone marrow (BM)‐MSCs in vitro. A, Flow cytometric gating strategy for identifying IL‐10 producing transitional B cells (CD19^+^ CD27^−^ CD38^hi^ CD24^hi^ IL‐10^+^). B, tSNE plots of IL‐10^+^ transitional B subset (five healthy donors) identified in experimental conditions as denoted, with overlay of manually gated IL‐10^+^ cells (navy blue) on transitional B cells (light blue); and C, pooled data. D, Flow cytometric gating strategy for identifying CD19^+^ CD27^+^ CD24^hi^ IL‐10^+^ B cells. E, tSNE plots of IL‐10‐producing CD19^+^ CD24^hi^ CD27^+^ B subset (five healthy donors) identified in experimental conditions as denoted, with overlay of manually gated IL‐10^+^ cells (navy blue) on CD19^+^ CD27^+^ CD24^hi^ B cells (green); and F, pooled data. G, Flow cytometric gating strategy for identifying IL‐10^+^ plasmablasts (CD19^+^ CD27^+^ CD38^hi^ IL‐10^+^). H, tSNE plots of IL‐10^+^ plasmablast subset (five healthy donors) identified in experimental conditions as denoted, with overlay of manually gated IL‐10^+^ cells (navy blue) on plasmablast B cells (red); and I, pooled data. tSNE plots (B,E,H) were generated by concatenation of individual samples. tSNE map of all samples (left‐most map) were generated from combining sample files of unstimulated resting B, stimulated B, coculture with BM‐MSCs (three donors), and coculture with P‐MSCs (three donors). tSNE analysis was run on 3000 live CD19^+^ single cells per sample using five markers: CD19, CD27, CD38, CD24, and IL‐10. All samples = 120 000 events; individual resting B and stimulated B samples = 15 000 events (n = 5); individual MSCs cocultured samples = 45 000 events (n = 15). PB, plasmablasts; TR, transitional B. Data are shown as mean ± SD. *P < .05; **P < .01; ***P < .001

IL‐10‐producing regulatory B cells (Bregs) are a more recently discovered type of B cell which are immunomodulatory and critical in regulating immune responses involved in inflammation, autoimmunity, and cancer.^9^ Major subsets of human IL‐10‐producing B cells can be found within the transitional B subset (CD19^+^ CD27^−^ CD38^hi^ CD24^hi^) and the CD27^+^ CD24^hi^ subset, with this latter Breg subset reported to be higher in women with normal pregnancies.^37^, ^38^, ^39^ We therefore assessed whether MSCs can induce immunomodulatory B cells in both subsets. We found that although coculture of stimulated B cells with BM‐MSCs can maintain transitional Breg percentages (gating strategies for outlined in Figure 3A), coculture with P‐MSCs significantly increased these percentages by approximately 40% (Figure 3B, tSNE plot; Figure 3C, pooled results; and Figure S3A, representative flow cytometric dot plots). For the CD27^+^ CD24^hi^ B‐cell subset (gating strategies outlined in Figure 3D), we found that coculture of stimulated B cells with P‐MSCs but not BM‐MSCs significantly increased IL‐10‐producing cell percentages (Figure 3E, tSNE plot; Figure 3F, pooled results; and Figure S3B, representative flow cytometric dot plots). IL‐10‐producing plasmablasts have also been identified recently^38^, ^40^ (gating strategies outlined in Figure 3G), and similar to the results found with transitional Bregs, we found that although coculture of stimulated B cells with BM‐MSCs maintained the levels of plasmablast Bregs, coculture with P‐MSCs significantly increased these Bregs by approximately 37% (Figure 3H, tSNE plot; Figure 3I, pooled results; and Figure S3C, representative flow cytometric dot plots). These findings demonstrate that P‐MSCs significantly increase multiple populations of IL‐10‐producing Bregs—including in transitional B, CD27^+^ CD24^hi^ B, and plasmablast subsets—in stimulated peripheral B cells.

### 3.3 P‐MSCs but not BM‐MSCs significantly inhibit stimulated B‐cell proliferation/activation and increase transitional B‐cell subset percentage in vivo

**FIGURE 4:**
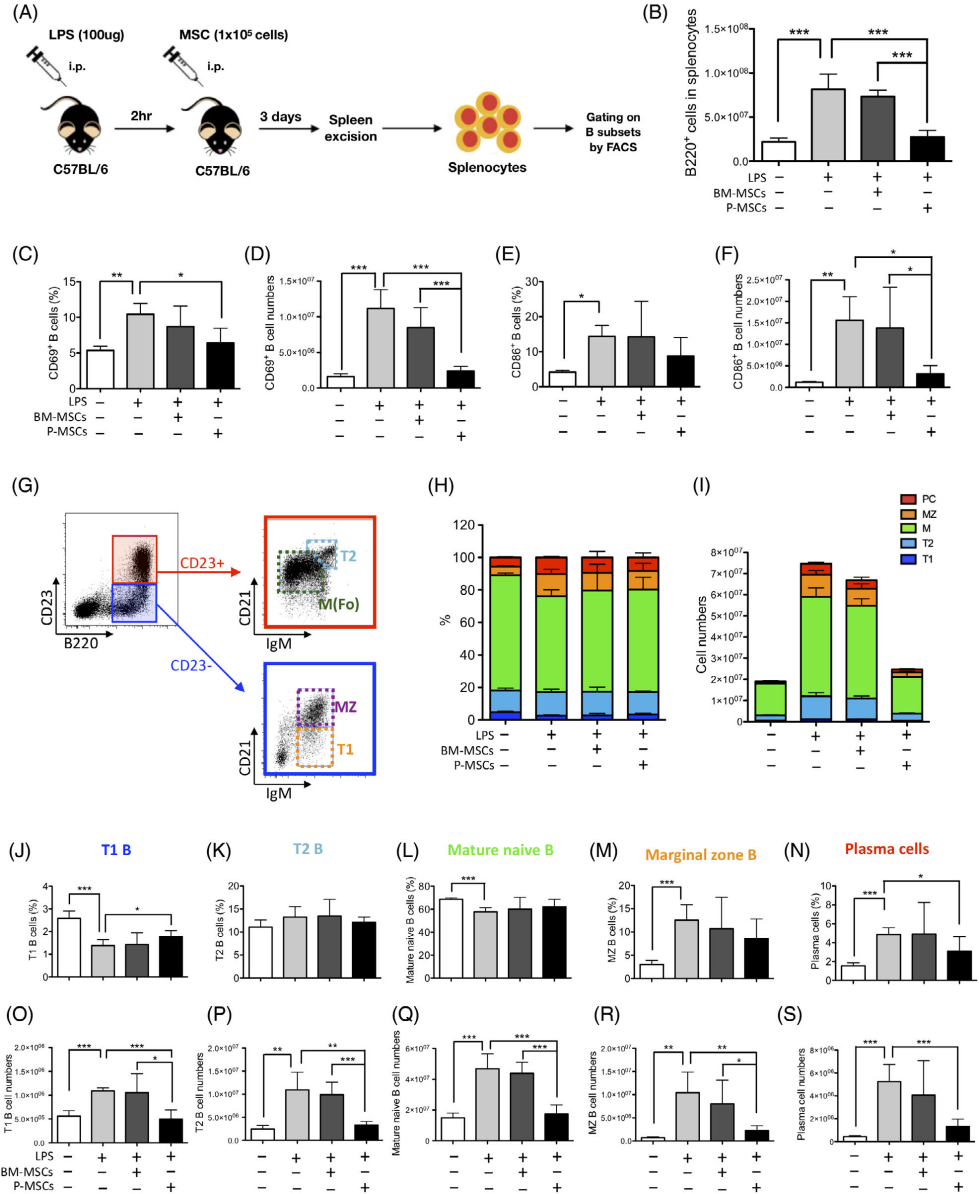
Placenta mesenchymal stromal cells (P‐MSCs) but not bone marrow (BM)‐MSCs significantly inhibit stimulated B‐cell proliferation/activation and increase transitional B‐cell subset percentages in vivo. A, Experimental schema of in vivo B‐cell activation using lipopolysaccharide (LPS) with adoptive transfer of hMSCs. Mice were intraperitoneally (i.p.) treated with LPS (100 μg/mouse), with i.p. injection of 1 × 10^5^ of BM‐MSCs or P‐MSCs 2 hours after. Mice were sacrificed 3 days later, with spleens excised for analysis of B‐cell populations. B, B220^+^ B‐cell numbers of LPS‐challenged mice without and with adoptive transfer of either human BM‐ or P‐MSCs. C, Cell percentages and, D, absolute cell numbers of CD69^+^ activated peripheral B220^+^ B cells; n = 5 in each experimental group. E, Cell percentages and, F, absolute cell numbers of CD86^+^ activated peripheral B220^+^ B cells; n = 5 in each experimental group. G, Flow cytometric gating strategy for identifying B‐cell subsets including transitional 1 B (T1B; B220^+^ CD23^−^ IgM^hi^ CD21^int^), transitional 2 B (T2B; B220^+^ CD23^+^ IgM^hi^ CD21^hi^), mature naive B (B220^+^ CD23^+^ IgM^−/int^ CD21^int^), and MZ B (MZ B; B220^+^ CD23^−^ IgM^hi^ CD21^hi^) subsets. H, Integrated data of B‐cell subset percentages. I, Integrated data of absolute B‐cell numbers. Each B subset percentages and absolute cell number are presented, respectively, as follows: J,O, transitional 1 B; K,P, transitional 2 B; L,Q, mature naive B; M,R, MZ B; and N,S, plasma cell. M, mature naive B; MZ, marginal zone B; PC, plasma cell; T1, transitional 1 B; T2, transitional 2 B. All percentage figures are gated on B220^+^ cells. Data are shown as mean ± SD. *P < .05; **P < .01; ***P < .001

To corroborate whether our in vitro findings using human peripheral pan‐B cells are physiologically relevant, we turned to an in vivo mouse model of B‐cell activation using LPS (Figure 4A, experimental schema), which can stimulate polyclonal B cells to proliferate and differentiate into functional plasma cells capable of secreting antibodies in vitro^41^ and in vivo.^42^ Compared to control/PBS‐injected mice, B220^+^ pan‐B cell numbers in LPS‐challenged mice were significantly increased 3.7‐fold, indicating that a strong immune response was elicited (Figure 4B). Strikingly, adoptive transfer of P‐MSCs to LPS‐challenged mice significantly reduced B220^+^ pan‐B cell absolute numbers down to unstimulated baseline levels; BM‐MSCs, however, had minimal effects on stimulated B‐cell numbers. Moreover, we found that adoptive transfer of P‐MSCs but not BM‐MSCs significantly suppressed the percentage as well as the absolute numbers of CD69^+^ activated peripheral B220^+^ B cells (Figure 4C, pooled percentages; Figure 4D, pooled absolute cell numbers; and Figure S4A, representative flow cytometric dot plots); P‐MSCs but not BM‐MSCs transfer also decreased the percentages of CD86^+^ activated peripheral B220^+^ B cells, and significantly decreased absolute cell numbers (Figure 4E, pooled percentages; Figure 4F, pooled absolute cell numbers; and Figure S4B, representative flow cytometric dot plots). Thus, P‐MSCs but not BM‐MSCs significantly suppressed stimulated B‐cell proliferation and activation in vivo.

We then analyzed the changes to specific peripheral B subsets after MSC treatment in vivo (Figure 4G, gating strategy). We found that adoptive transfer of P‐MSCs but not BM‐MSCs led to maintenance of the percentage of undifferentiated transitional 1 (T1) B cells (Figure 4J, pooled percentages; Figure S4C, representative flow cytometric dot plots; and Figure S5A, tSNE plots). Notably, in the mouse system, although T1B percentages were decreased after LPS stimulation (Figure 4J), the absolute cell numbers were significantly increased (Figure 4O) which is in contrast to the human in vitro system in which transitional B cells were significantly decreased in both percentage and absolute number after stimulation (Figure 2C,H). Despite these inherent differences, adoptive transfer of P‐MSCs but not BM‐MSCs in vivo significantly increased the percentage of T1B cells while decreasing its absolute numbers relative to results with BM‐MSCs (Figure 4J,O, respectively), similarly to the human in vitro data on transitional B subset (Figure 2C,H). For all other B‐cell subsets, there appears to be no significant differences in the percentage of transitional 2 (T2) B, mature naive B, and marginal zone (MZ) B cells in spleen after either BM‐ or P‐MSCs adoptive transfer (Figure 4H,K‐M). However, when absolute cell numbers are assessed, adoptive transfer of P‐MSCs but not BM‐MSCs decreased the absolute cell numbers of all B‐cell subsets, not only of T1B but also T2B, mature naive B, and MZ B cells as well (Figure 4I,O‐R). This trend was also seen with murine plasma cells, which are gated separately as B220^+^ CD138^hi^ B cells, with adoptive transfer of P‐MSCs but not BM‐MSCs significantly suppressing both the percentage and absolute number of plasma cells in vivo (Figure 4N, pooled percentages; Figure 4S, pooled absolute cell numbers; Figure S4D, representative flow cytometric dot plots; and Figure S5B, tSNE plots). Overall, the in vivo murine data strongly corroborate with the in vitro human data, implicating that P‐MSCs strongly suppress stimulated B‐cell proliferation and maintain these lymphocytes in a more early and undifferentiated state, whereas BM‐MSCs support proliferation and continued development/differentiation.

### 3.4 P‐MSCs induce more IL‐10‐producing Breg subsets than BM‐MSCs in vivo

**FIGURE 5:**
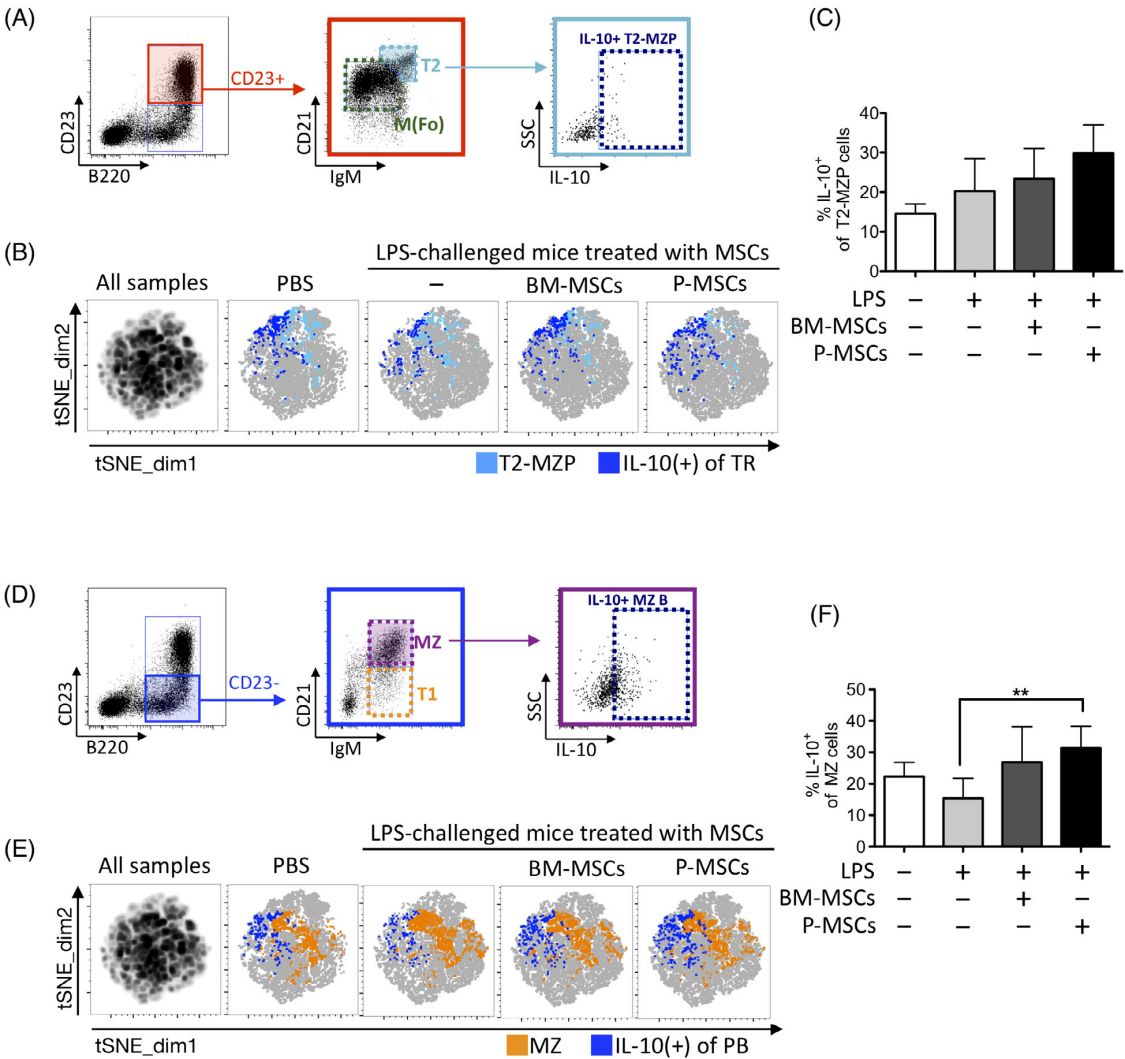
Placenta mesenchymal stromal cells (P‐MSCs) induce more IL‐10‐producing Breg subsets than bone marrow (BM)‐MSCs in vivo. A, Flow cytometric gating strategy for identifying IL‐10 producing transitional 2‐marginal zone precursor (T2‐MZP) subset (CD19^+^ CD23^+^ IgM^hi^ CD21^hi^ IL‐10^+^). B, tSNE plots of IL‐10^+^ T2‐MZP subset (n = 5 in each group) identified in experimental conditions as denoted, with overlay of manually gated IL‐10^+^ cells (navy blue) on T2‐MZP (light blue); and C, pooled data. D, Flow cytometric gating strategy for identifying IL‐10 producing MZ B subset (CD19^+^ CD23^−^ IgM^hi^ CD21^hi^ IL‐10^+^). E, tSNE plots of IL‐10^+^ MZ B subset (n = 5 in each group) identified in experimental conditions as denoted, with overlay of manually gated IL‐10^+^ cells (navy blue) on MZ B cells (orange); and F, pooled data. tSNE plots (B,E) were generated by concatenation of individual samples. tSNE map of all samples (left‐most map) were generated from combining sample files of PBS control, lipopolysaccharide (LPS)‐challenged, BM‐MSC‐, and P‐MSC‐adoptive transfers. tSNE analysis was run on 6000 live CD19^+^ single cells per sample using five markers: CD19, CD23, IgM, CD21, and IL‐10. All samples = 120 000 events; individual PBS control and LPS‐challenged samples = 30 000 events (n = 5); individual MSCs adoptive transfer samples = 30 000 events (n = 5). Data are shown as mean ± SD. *P < .05; **P < .01; ***P < .001

Our in vitro human data demonstrated that P‐MSCs are more potent than BM‐MSCs at induce multiple IL10‐producing Breg subsets (Figure 3). To corroborate whether this in vitro data had in vivo relevance, we assessed in the murine model for IL‐10 production first in the T2B subset (also called transitional 2‐marginal zone precursor, T2‐MZP), in which Bregs have been most commonly identified.^43^ We found that after LPS stimulation in vivo, while statistical significance was not reached, adoptive transfer of P‐MSCs compared to BM‐MSCs induced more IL‐10^+^ T2‐MZP cells (Figure 5A, gating strategies; Figure 5B, tSNE plot; Figure 5C, pooled results; and Figure S6A, representative flow cytometric dot plots). In addition to T2‐MZP cells, IL10‐producing Bregs has also been identified in the MZ B cell subset.^44^ Interestingly, although both P‐ and BM‐MSCs can increase levels of IL‐10^+^ MZ B cells (Figure 5D, gating strategies), only P‐MSCs induced a significant increase of these immunomodulatory B cells (Figure 5E, tSNE plot; Figure 5F, pooled results; and Figure S6B, representative flow cytometric dot plots). Thus, these findings suggest that P‐MSCs increase IL‐10‐producing cells in T2‐MZP and significantly in MZ B subsets of LPS‐challenged mice.

### 3.5 Differential interactions of BM‐ and P‐MSCs globally on multiple B‐cell subsets are multifactorial

**FIGURE 6:**
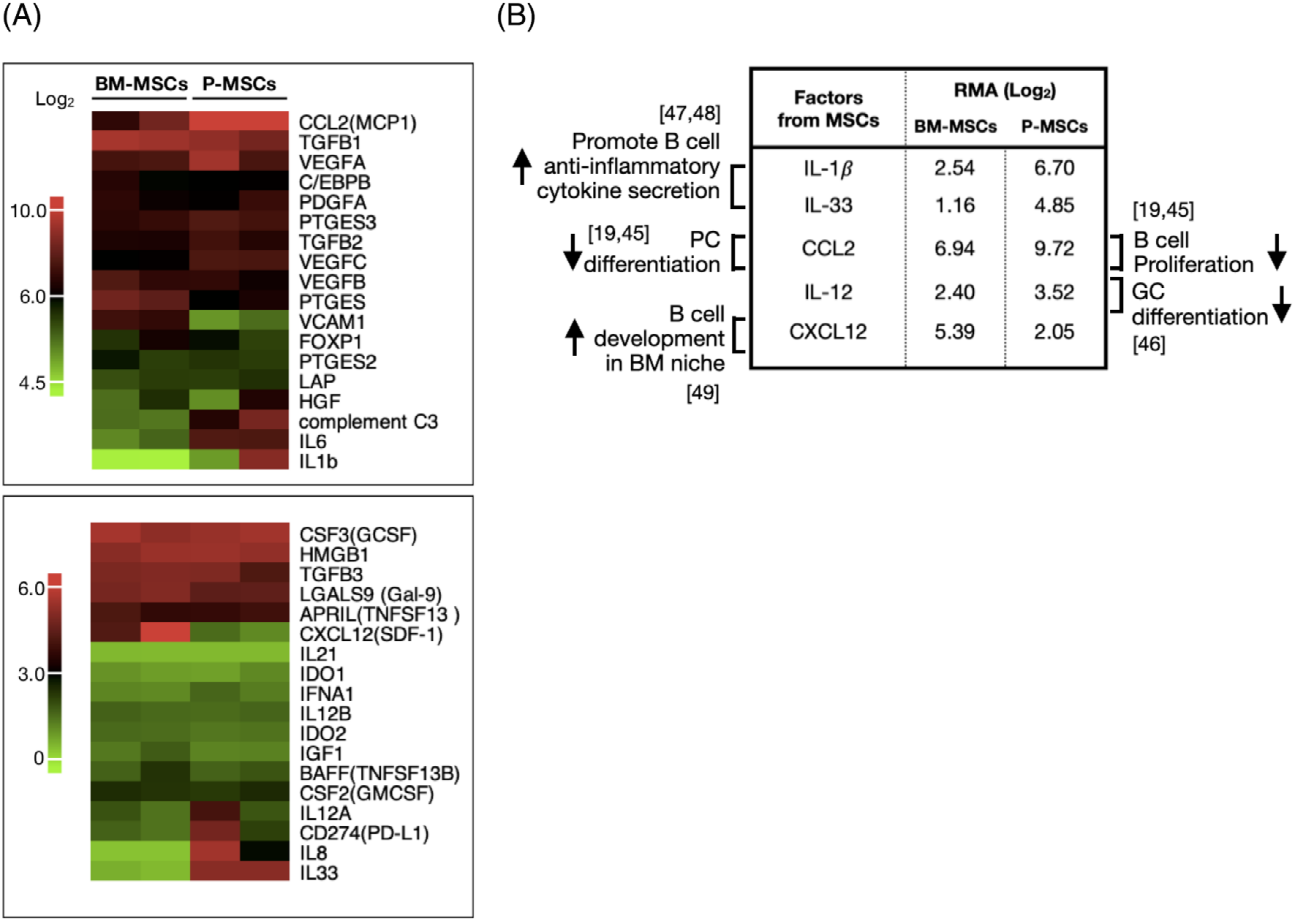
Differential interactions of bone marrow (BM‐) and placenta mesenchymal stromal cells (P‐MSCs) globally on multiple B‐cell subsets are multifactorial. A, Heatmap of immunomodulatory factors relevant to B‐cell modulation as expressed by BM‐MSCs and P‐MSCs (2 donors each). Top heatmap, range of scale robust multi‐array (RMA) values: 4.5 to 10; bottom heatmap range: 0 to 6. B, Table detailing BM‐ and P‐MSC expression levels of the most relevant factors mediating observed effects on B‐cell proliferation and differentiation, with citation of relevant references. GC, germinal center; PC, plasma cells

**FIGURE 7:**
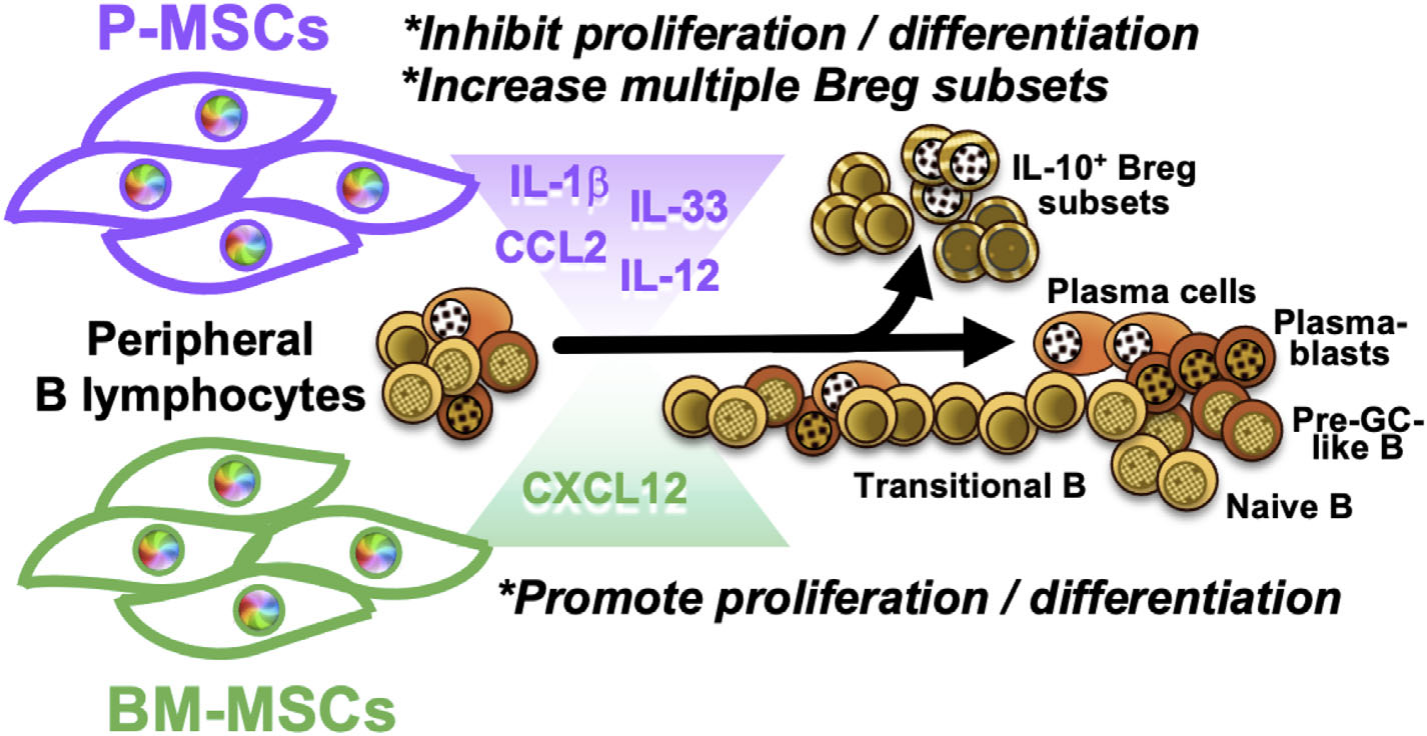
B lymphocyte interactions with resident bone marrow (BM) mesenchymal stromal cells (MSCs) vs nonresident placental MSCs (P‐MSCs) result in disparate outcomes. BM‐MSCs supported but P‐MSCs suppressed stimulated peripheral B‐cell proliferation and further differentiation, whereas P‐MSCs more significantly increased multiple populations of IL‐10‐expressing regulatory B cells (Bregs). Multifactorial differences in expression of relevant factors by the two sources of MSCs are likely involved in these divergent outcomes

To identify relevant factors expressed by either MSCs which are involved in the divergent B‐cell interactions, we performed transcriptome profiling on both types of MSCs and compiled a list of possible factors influencing B‐cell proliferation and differentiation based on literature search (Figure 6A). Overall analyses revealed that P‐MSCs express numerous factors that inhibit B‐cell proliferation, plasma cell differentiation, and germinal center differentiation, as well as promote anti‐inflammation cytokines secretion, at a higher level than BM‐MSCs (Figure 6B). Notably, P‐MSCs express CCL2, a factor involved in decreasing B‐cell proliferation and plasma cell differentiation^19^, ^45^ at higher levels (1.4‐fold) than BMMSCs. P‐MSCs also express higher levels of IL‐12 (1.5‐fold) which has been reported to inhibit germinal center differentiation.^46^ Moreover, P‐MSCs express IL‐1𝛽 and IL‐33, two factors that have been reported to promote B‐cell anti‐inflammatory cytokine production,^47^, ^48^ at two‐ to fourfold levels higher than BM‐MSCs. On the other hand, BM‐MSCs express twice the level of CXCL12, a factor that maintains B‐cell development within the BM niche,^49^ than P‐MSCs. Thus, the differential interactions of BM‐ and P‐MSCs globally on peripheral B‐cell proliferation and differentiation/maturation appear multifactorial in origin (Figure 7).

## 4 DISCUSSION

Despite the wide use of MSCs for immune and inflammatory diseases, there is limited understanding in the interactions of these stromal cells with B lymphocytes, which are critically involved in a wide range of autoimmune and other immune‐related diseases. Moreover, studies are emerging on functional differences between tissue‐specific MSCs. We demonstrate in both in vitro and in vivo settings that diverse sources of human MSCs exert markedly different interactions with stimulated peripheral B lymphocytes, with BM‐MSCs—the MSCs native to the site of B cell origin—supporting proliferation and continued differentiation including maintaining IL‐10‐producing Breg subsets. In contrast, P‐MSCs significantly suppress stimulated peripheral B‐cell proliferation to the extent to halt further differentiation, while significantly increasing multiple IL‐10‐producing Breg subsets. Transcriptome analyses of BM‐ and P‐MSCs revealed different profiles of relevant factors affecting B lymphocyte proliferation and differentiation which could contribute to the divergent outcome that we found, but further investigation is required for mechanistic validation of factor‐specific contributions.

Our findings on the overall supportive effect of BM‐MSCs on stimulated peripheral B‐cell growth and continued differentiation are not surprising given that this is the resident MSCs from the niche for B‐cell origin and initial development. Regarding the strong suppressive effects of P‐MSCs on B‐cell growth and differentiation, this may be due to the placenta being a highly immunomodulatory organ. As IL‐10 is critical for optimal reproduction,^50^, ^51^, ^52^ Bregs may be particularly important during pregnancy. Recent studies demonstrate that mouse placental explant (from pregnant‐day 9 mice) cocultured with murine B cells upregulate IL‐10 production,^39^ and peripheral blood IL‐10‐producing CD19^+^CD24^hi^CD27^+^ B cells are significantly higher in women with normal pregnancies compared to nonpregnant women or women who have had spontaneous abortions.^53^ Although few, such reports are beginning to reveal not only the important role of IL‐10‐producing Bregs during pregnancy, but that the placenta may be a specialized niche for promoting these immunomodulatory B cells,^54^ which our findings of P‐MSCs significantly inducing multiple subsets of Bregs support. In keeping with the known immunomodulation of P‐MSCs toward T cells, NKs, and myeloid cells, our findings here further extend those immunomodulatory properties toward peripheral B cells, with significant induction of multiple populations of IL‐10‐producing Bregs compared to BM‐MSCs. Indeed, an increasing number of studies show that fetal‐source umbilical cord‐MSCs suppress in vitro B‐cell proliferation and halt terminal differentiation^55^, ^56^; two very recent reports using amniotic membrane‐MSCs demonstrate similar findings,^57^ and decreased pulmonary B‐cell recruitment and maturation was found in an in vivo lung injury model.^58^ Moreover, our lab and others have found that fetal‐source MSCs^29^, ^59^—in contrast to adult BM‐MSCs^60^—may not require inflammatory priming for immunomodulation, an aspect that has not been evaluated for MSC interactions with B cells. Our study reveal the highly divergent and comprehensive modulation of pan‐B lymphocytes by MSCs from different origins, which may partially explain why in vivo and clinical outcomes of human MSC therapy has not been as robust as expected, especially in immune‐related diseases now known to have a strong B‐cell presence.^10^, ^11^ In fact, the most efficacious outcome of MSC clinical trials has been for T cell‐mediated diseases especially those with strong Th17 presence^3^; therefore, our findings may have translational relevance in selecting optimal MSC sources for diseases with strong B‐cell presence and/or less dominant T‐cell presence. Curiously, despite the availability of so many sources MSCs for clinical use, functional head‐to‐head studies evaluating efficacy of different sources of MSCs have been rare. Clearly, such investigations on specific functional properties of different sources of MSCs in a global and comprehensive fashion are urgently needed for improving clinical efficacy of MSC therapy.

The current reports on MSC‐B cell interactions have yielded discrepant conclusions, even if only reports using BM‐MSCs—the most commonly used source—are considered.^12^ One reason for such disparate results is due to the differences in the activation/maturation protocols utilized, which ranged from TLR activation CpG only^61^ to activation cocktails with different combinations of anti‐CD40, F(ab)2 anti‐IgM, CpG, and cytokines (IL‐2 or/and IL‐4).^17^, ^19^, ^32^, ^62^ Such diverse methods are known to result in different outcomes for peripheral B‐cell differentiation/maturation.^12^ We chose a more standard protocol with an activation cocktail (F(ab)2 anti‐IgM, anti‐CD40, CpG, and IL‐2) to mimic antigen‐ and T cell‐mediated stimulation.^32^ In addition, the B‐cell population selected for study have also been inconsistent between reports, with reports using either positively selected CD19^+^ B cells^17^, ^19^, ^63^ or CD43^negative^ resting B cells,^32^ neither of which include terminal B subsets such as plasmablasts, or nonresting B cells such as pre‐GC‐like B, memory B, and plasmablasts, respectively. We utilized a negative selection process to isolate as many peripheral B‐cell populations as possible—including naive, memory B cells, and plasmablasts—to allowed for a more complete picture of MSC B‐cell interactions. We were therefore able to observe global and complex changes in multiple peripheral B‐cell populations simultaneously after interaction with each MSC source, as well as compare these changes between BM‐ and P‐MSC interactions, to provide the most comprehensive evaluation to date on this topic.

## 5 CONCLUSION

Our findings overall demonstrate that diverse sources of MSCs harbor different interactions with peripheral B lymphocytes, with BM‐MSCs—the MSC resident to B‐cell origin and developmental niche—supporting overall stimulated B‐cell proliferation and allowing for more terminal differentiation into effector cell types than P‐MSCs, which exert profound suppressive effects on B‐cell proliferation and terminal effector differentiation while significantly increasing multiple IL10‐producing Breg populations. These divergent interactions between BM‐ and P‐MSCs with B cells could be due to the different profile of multiple relevant factors expressed by these two sources of MSCs. Our results not only highlight the differential and global interactions of diverse human MSC sources with peripheral B lymphocytes, but also demonstrate the importance of understanding MSC tissue‐specific differences to achieve more effective therapeutic outcome.

## CONFLICT OF INTEREST

The authors declared no potential conflicts of interest.

## AUTHOR CONTRIBUTIONS

W.L.: designed the research, performed experiments, analyzed the data, and wrote the manuscript; L.T.W., M.L.Y., P.J.H., Y.W.L.: performed experiments and analyzed data; K.J.L., K.I.L., Y.W.S., H.K.S.: analyzed data and provided feedback; B.L.Y.: conceived the idea, oversaw the research, revised the manuscript, and provided funding.

## Supporting information


**Supplementary Figure 1** **Time line of stimulated in vitro peripheral human B cell differentiation**. Pan‐B cells isolated from human PBMCs (3 healthy donors) were stimulated with anti‐Ig, CD40L, IL‐2 and CpG‐ODNs (detailed isolation and stimulation protocol described in Materials & Methods section) and cultured in vitro up to 7 days, with flow cytometric analyses from the third to seventh day to determine the percentage of IgG^+^ (intracellular staining) plasmablasts. (A) Gating strategy for IgG^+^ plasmablasts. (B) Representative IgG^+^ plasmablasts flow cytometric dot plots. (C) Pooled flow cytometric data for IgG^+^ plasmablasts.
**Supplementary Figure 2: P‐MSCs suppress the proliferation of all B cell subsets more strongly than BM‐MSCs**. (A) Representative data and (B) pooled data of cell proliferation for each B subsets (3 healthy donors) as assessed by CFSE staining, then co‐culture with either BM‐ or P‐MSCs to calculate B cells undergoing proliferation by flow cytometric analyses. Data are shown as mean ± SD. *, *P* < 0.05; **, *P* < 0.01; ***, *P* < 0.001.
**Supplementary Figure 3: Representative flow cytometry dot plot for IL‐10 intracellular staining in human B cell subsets:** (A) transitional B cells (CD19^+^ CD27^−^ CD38^hi^ CD24^hi^ IL‐10^+^), (B) CD19^+^ CD27^+^ CD24^hi^ IL‐10^+^ B cells, and (C) plasmablasts (CD19^+^ CD27^+^ CD38^hi^ IL‐10^+^).
**Supplementary Figure 4: P‐MSCs but not BM‐MSCs significantly inhibit stimulated B cell proliferation/activation and increase transitional B cell subset percentages in vivo**. (A) Representative data of CD69^+^ activated peripheral B220^+^ B cells; n = 5 in each experimental group. (F) Representative data of CD86^+^ activated peripheral B220^+^ B cells; n = 5 in each experimental group. (C) Representative data for B cell subset analyses in resting B cells, and LPS‐challenged B cells co‐cultured without or with either BM‐MSCs or P‐MSCs; n = 5 in each experimental group. (D) Representative data for plasma cell (B220^+^ CD138^+^) subset analyses in resting B cells, and LPS‐challenged B cells co‐cultured without or with either BM‐MSCs or P‐MSCs; n = 5 in each experimental group.
**Supplementary Figure 5: P‐MSCs but not BM‐MSCs increase transitional B cell subset percentages in vivo**. tSNE plots for (A) all B cell subsets except plasma cells, with concatenated flow cytometry standard files for all samples (left‐most map), and individual resting B cells, and LPS‐challenged B cells co‐cultured without or with either BM‐MSCs or P‐MSCs. Overlay of manually gated cell populations on to tSNE plots. B cell subsets are identified using flow cytometric biaxial gating strategies based on 4 surface markers into transitional 1 B (T1B; B220^+^ CD23^−^ IgM^hi^ CD21^int^), transitional 2 B (T2B; B220^+^ CD23^+^ IgM^hi^ CD21^hi^), mature naive B (M; B220^+^ CD23^+^ IgM^−/int^ CD21^int^), and MZ B (MZ; B220^+^ CD23^−^ IgM^hi^ CD21^hi^) subsets. (B) tSNE plots for B220^+^ CD138^+^ plasma cells (PC), with concatenated flow cytometry standard files for all samples (left‐most map), and individual resting B cells, and LPS‐challenged B cells co‐cultured without or with either BM‐MSCs or P‐MSCs. Overlay of manually gated cell populations on to tSNE plots. Plasma cells are identified using flow cytometric biaxial gating strategies based on positivity for both surface markers: B220 and CD138. tSNE analysis was performed on 6000 live B220^+^ single cells per sample. All samples = 120 000 events; individual PBS and LPS samples = 30 000 events (n = 5); individual MSCs adoptive transfer samples = 30 000 events (n = 5). Each experimental group consisted of 5 mice.
**Supplementary Figure 6: Representative flow cytometry dot plot for IL‐10 intracellular staining in mouse B cell subsets:** (A) T2‐MZP subset (CD19^+^ CD23^+^ IgM^hi^ CD21^hi^ IL‐10^+^), and (B) MZ B subset (CD19^+^ CD23^−^ IgM^hi^ CD21^hi^ IL‐10^+^).

**Supplementary Table 1** Immunophenotypes of assessed human and murine B cell subsets.

## Data Availability

The data that support the findings of this study are available from the corresponding author upon reasonable request.
