# Supplementary material for: Resident vs nonresident multipotent mesenchymal stromal cell interactions with B lymphocytes result in disparate outcomes
Source: Stem Cells Transl Med. 2021 Jan 28;10(5):711–24. doi: 10.1002/sctm.20-0289 (PMC8046079; doi:10.1002/sctm.20-0289)

Supplementary Figure 1

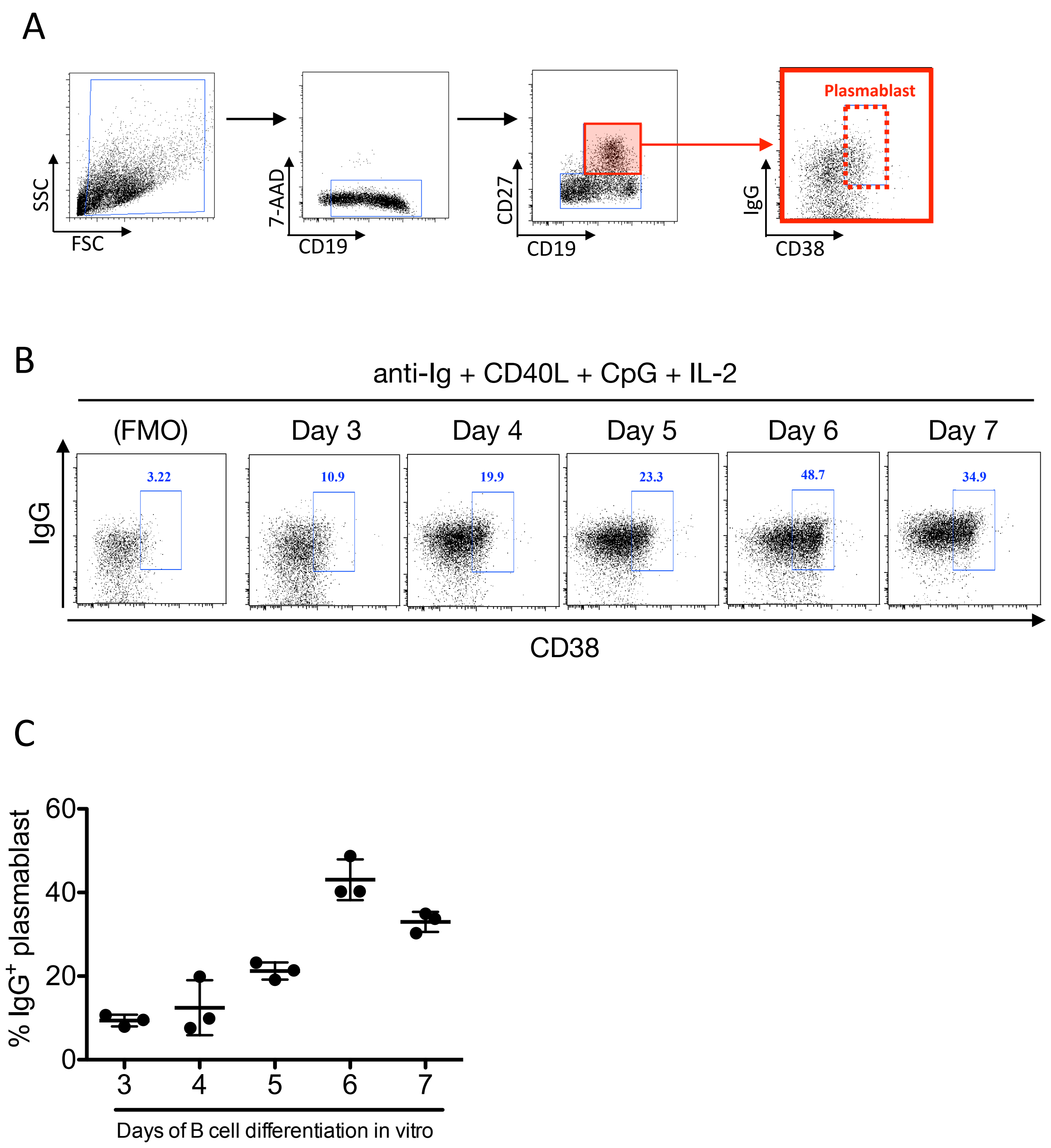

Supplementary Figure 2

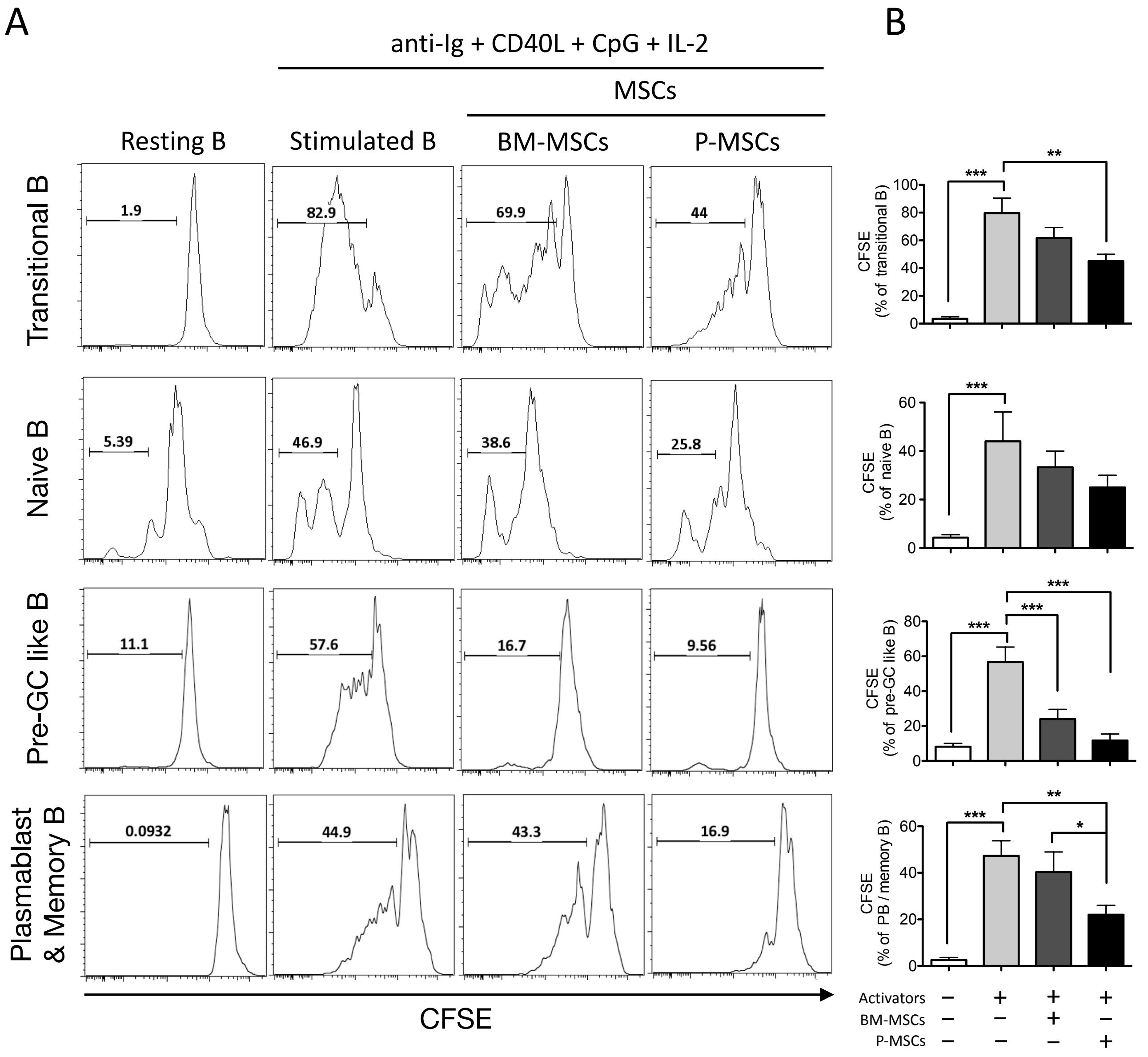

Supplementary Figure 3

A **Transitional B cells ($CD19^{+}$ $CD27^{-}$ $CD38^{hi}$ $CD24^{hi}$ IL-10^{+})**

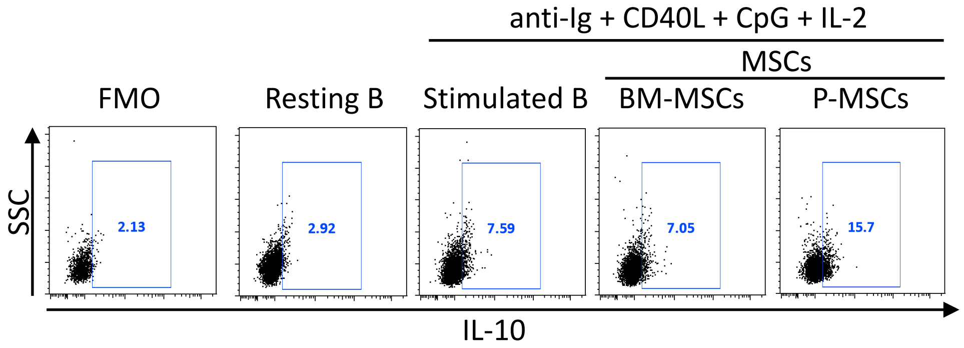

B **$CD19^{+}$ $CD27^{+}$ $CD24^{hi}$ IL-10^{+} B cells**

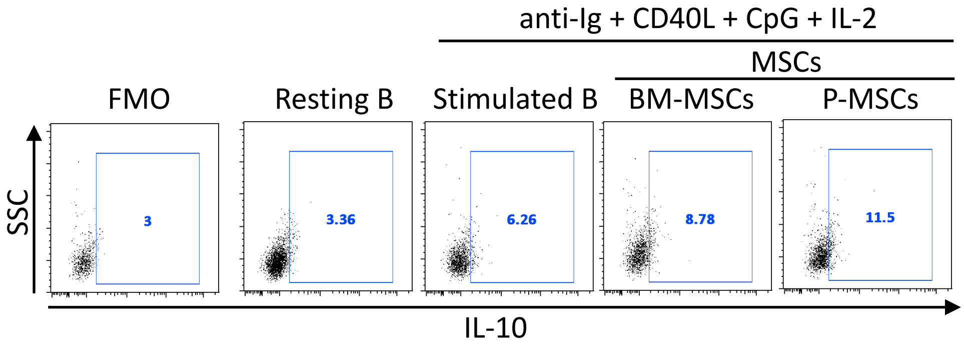

C **Plasmablasts ($CD19^{+}$ $CD27^{+}$ $CD38^{hi}$ IL-10^{+})**

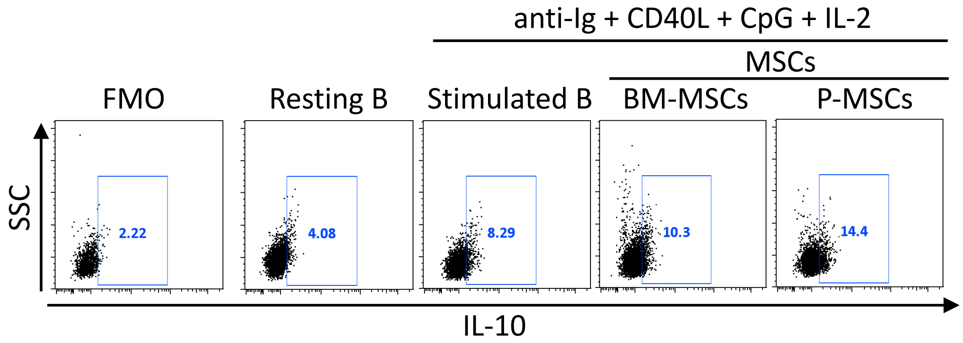

Supplementary Figure 4

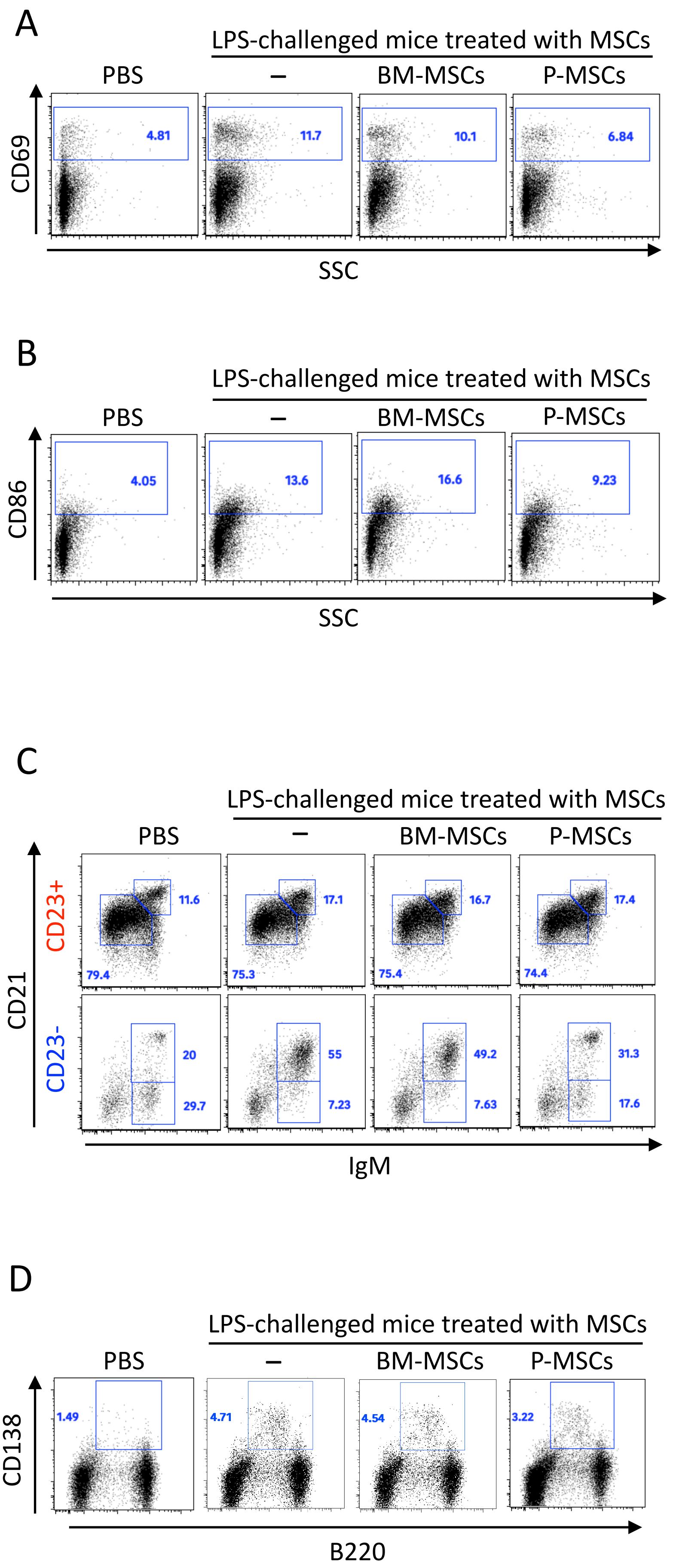

Supplementary Figure 5

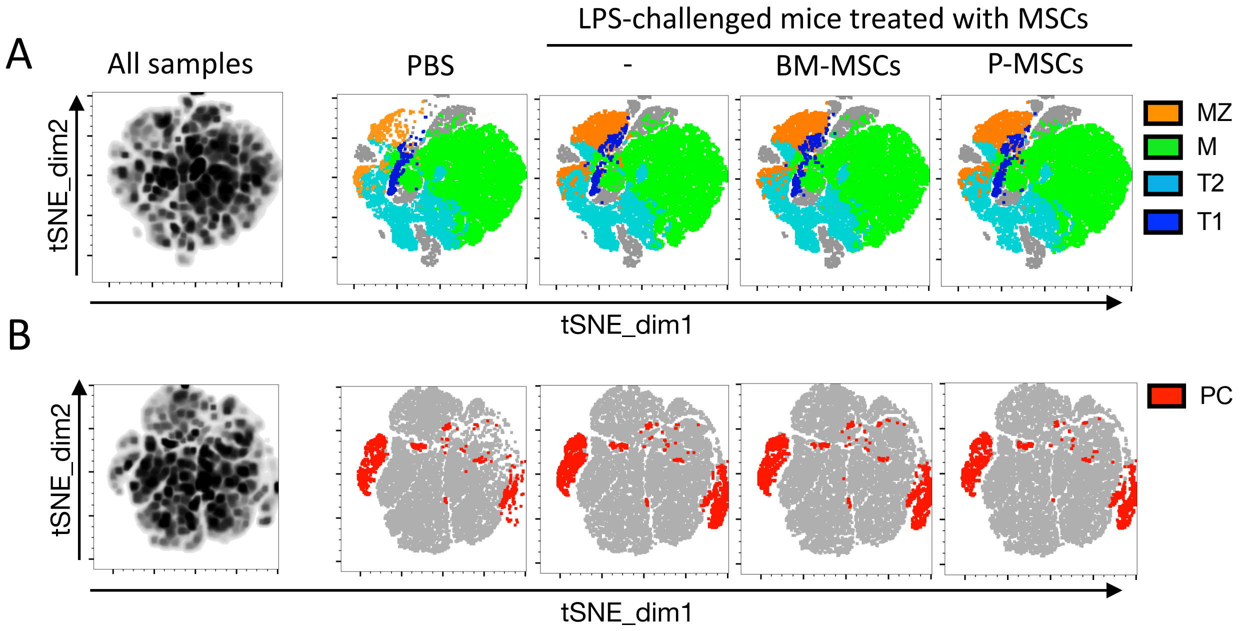

Supplementary Figure 6

A **T2-MZP subset (CD19^{+} CD23^{+} IgM^{hi} CD21^{hi} IL-10^{+})**

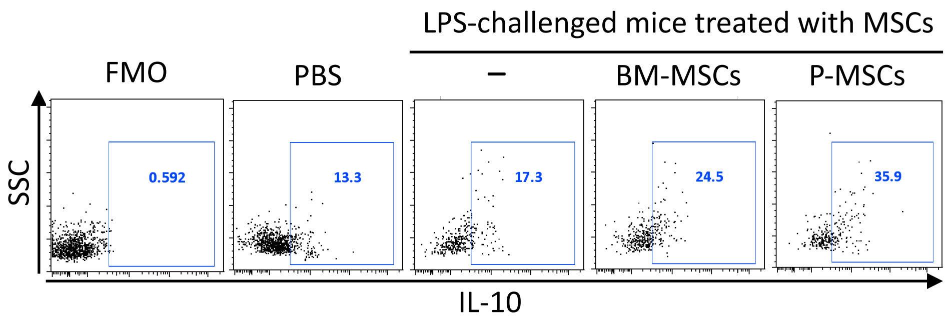

B **MZ B subset (CD19^{+} CD23^{-} IgM^{hi} CD21^{hi} IL-10^{+})**

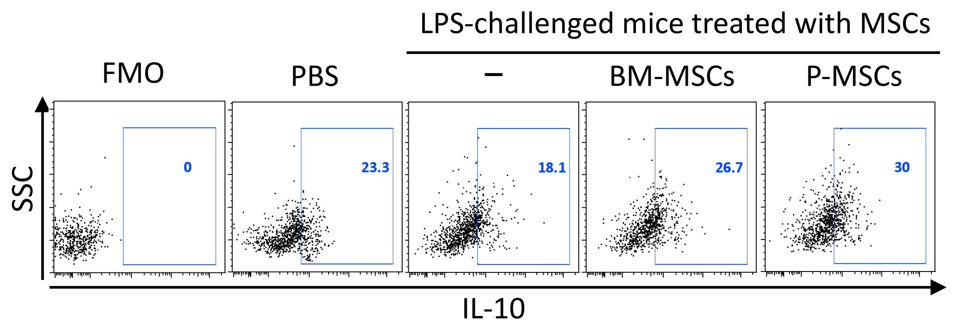

Supplement: Supplementary file 1 — Supplementary Figure 1 Time line of stimulated in vitro peripheral human B cell differentiation. Pan‐B cells isolated from human PBMCs (3 healthy donors) were stimulated with anti‐Ig, CD40L, IL‐2 and CpG‐ODNs (detailed isolation and stimulation protocol described in Materials & Methods section) and cultured in vitro up to 7 days, with flow cytometric analyses from the third to seventh day to determine the percentage of IgG+ (intracellular staining) plasmablasts. (A) Gating strategy for IgG+ plasmablasts. (B) Representative IgG+ plasmablasts flow cytometric dot plots. (C) Pooled flow cytometric data for IgG+ plasmablasts. Supplementary Figure 2: P‐MSCs suppress the proliferation of all B cell subsets more strongly than BM‐MSCs. (A) Representative data and (B) pooled data of cell proliferation for each B subsets (3 healthy donors) as assessed by CFSE staining, then co‐culture with either BM‐ or P‐MSCs to calculate B cells undergoing proliferation by flow cytometric analyses. Data are shown as mean ± SD. *, P < 0.05; **, P < 0.01; ***, P < 0.001. Supplementary Figure 3: Representative flow cytometry dot plot for IL‐10 intracellular staining in human B cell subsets: (A) transitional B cells (CD19+ CD27− CD38hi CD24hi IL‐10+), (B) CD19+ CD27+ CD24hi IL‐10+ B cells, and (C) plasmablasts (CD19+ CD27+ CD38hi IL‐10+). Supplementary Figure 4: P‐MSCs but not BM‐MSCs significantly inhibit stimulated B cell proliferation/activation and increase transitional B cell subset percentages in vivo. (A) Representative data of CD69+ activated peripheral B220+ B cells; n = 5 in each experimental group. (F) Representative data of CD86+ activated peripheral B220+ B cells; n = 5 in each experimental group. (C) Representative data for B cell subset analyses in resting B cells, and LPS‐challenged B cells co‐cultured without or with either BM‐MSCs or P‐MSCs; n = 5 in each experimental group. (D) Representative data for plasma cell (B220+ CD138+) subset analyses in resting B cells, and LPS‐chall [file SCT3-10-711-s001.pdf]
