# Supplementary material for: Resident vs nonresident multipotent mesenchymal stromal cell interactions with B lymphocytes result in disparate outcomes
Source: Stem Cells Transl Med. 2021 Jan 28;10(5):711–24. doi: 10.1002/sctm.20-0289 (PMC8046079; doi:10.1002/sctm.20-0289)
Supplement: Supplementary file 2 — Supplementary Table 1 Immunophenotypes of assessed human and murine B cell subsets. [file SCT3-10-711-s002.docx]

**Supplementary Table 1**. Immunophenotypes of assessed human and murine B cell subsets.

| **Cell population** | **Phenotype** | **Reference(s)** |
| --- | --- | --- |
| **HUMAN** |  |  |
| Transitional B cell | CD19^+^ CD27^-^ CD38^hi^ CD24^hi^ | [32, 64] |
| Naive B cell | CD19^+^ CD27^-^ CD38^-/int^ CD24^-/int^ | [32, 64] |
| Pre-GC-like B cell | CD19^+^ CD27^-^ CD38^hi^ CD24^-/int^ | [65] |
| Plasmablast & memory B cell | CD19^+^ CD27^+^ | [32, 64] |
| Plasmablast | CD19^+^ CD27^+^ CD38^hi^ | [32] |
| IL-10^+^ transitional B cell | CD19^+^ CD27^-^ CD38^hi^ CD24^hi^ IL-10^+^ | [32] |
| IL-10^+^ CD27^+^ CD24^hi^ B cell | CD19^+^ CD27^+^ CD24^hi^ IL-10^+^ | [37-39] |
| IL-10^+^ plasmablast cell | CD19^+^ CD27^+^ CD38^hi^ IL-10^+^ | [38, 40] |
|  |  |  |
| **MOUSE** |  |  |
| Transitional 1 B cell | B220^+^ CD23^-^ IgM^hi^ CD21^int^ | [66] |
| Transitional 2 B cell | B220^+^ CD23^+^ IgM^hi^ CD21^hi^ | [66] |
| Mature naive (follicular) B cell | B220^+^ CD23^+^ IgM^-/int^ CD21^int^ | [43, 66] |
| Marginal zone B cell | B220^+^ CD23^-^ IgM^hi^ CD21^hi^ | [43, 66] |
| Plasma cell | B220^+^ CD138^hi^ | [67] |
| IL-10^+^ T2-MZP B cell | B220^+^ CD23^+^ IgM^hi^ CD21^hi^ IL-10^+^ | [43] |
| IL-10^+^ marginal zone B cell | B220^+^ CD23^-^ IgM^hi^ CD21^hi^ IL-10^+^ | [44] |

Abbreviations: GC, germinal center; MZP, marginal zone precursor.
